# The Beneficial Impact of Antidepressant Drugs on Prenatal Stress-Evoked Malfunction of the Insulin-Like Growth Factor-1 (IGF-1) Protein Family in the Olfactory Bulbs of Adult Rats

**DOI:** 10.1007/s12640-015-9575-3

**Published:** 2015-11-26

**Authors:** Ewa Trojan, Katarzyna Głombik, Joanna Ślusarczyk, Bogusława Budziszewska, Marta Kubera, Adam Roman, Władysław Lasoń, Agnieszka Basta-Kaim

**Affiliations:** Department of Experimental Neuroendocrinology, Institute of Pharmacology, Polish Academy of Sciences, 12 Smętna St, 31-343 Cracow, Poland; Department of Brain Biochemistry, Institute of Pharmacology, Polish Academy of Sciences, 12 Smętna St, 31-343 Cracow, Poland

**Keywords:** Antidepressant drugs, Prenatal stress, Olfactory bulbs, Insulin-like-growth factor 1 (IGF-1) family

## Abstract

Insulin-like growth factor-1 (IGF-1) promotes the growth, differentiation, and survival of both neurons and glial cells, and it is believed to exert antidepressant-like activity. Thus, disturbances in the IGF-1 system could be responsible for the course of depression. To date, there have been no papers showing the impact of chronic antidepressant treatment on the IGF-1 network in the olfactory bulb (OB) in an animal model of depression. Prenatal stress was used as model of depression. Twenty-four 3-month-old male offspring of control and stressed mothers were subjected to behavioral testing (forced swim test). The mRNA expression of IGF-1 and IGF-1 receptor (IGF-1R) and the protein level of IGF-1 and its phosphorylation, as well as the concentrations of IGF-binding proteins (IGFBP-2, -4, -3, and -6), were measured in OBs before and after chronic imipramine, fluoxetine, or tianeptine administration. Adult rats exposed prenatally to stressful stimuli displayed not only depression-like behavior but also decreased IGF-1 expression, dysregulation in the IGFBP network, and diminished mRNA expression, as well as IGF-1R phosphorylation, in the OB. The administration of antidepressants normalized most of the changes in the IGF-1 system of the OB evoked by prenatal stress. These results suggested a beneficial effect of chronic antidepressant drug treatment in the alleviation of IGF-1 family malfunction in OBs in an animal model of depression.

## Introduction

Apart from changes in neurotransmitters and the dysregulation of the immune and endocrine systems, studies have postulated that impairments in synaptic plasticity and neurogenesis also play important roles in the development of depression (Bredt et al. 2015). Furthermore, data have shown that depression might result from alterations in the neurotrophic factors expression in the central nervous system (CNS) (Branchi et al. 2004). Interestingly, in addition to brain-derived neurotrophic factor (BDNF), insulin-like growth factor-1 (IGF-1) has recently attracted a significant amount of attention.

IGF-1 is a small peptide (7.5 kDa) that is produced not only in the periphery but also in various parts of the CNS, such as the cerebellum, hypothalamus, striatum, cerebral cortex, hippocampus, and olfactory bulbs (OB) (Bondy 1991; de Pablo and de la Rosa 1995). In the brain, IGF-1 has combined effects on neural cell signaling and neurotrophic responses (Hoshaw et al. 2008). Moreover, it plays a crucial role in brain development, mainly through control of cell growth, differentiation, maturation, and survival. Data have reported that IGF-1 exerts biological functions particularly via the IGF-1 receptor (IGF-1R), which is a transmembrane heterotetramer consisting of two extracellular α subunits containing the IGF-binding site and two intracellular β subunits that exhibit tyrosine kinase activity (Annuziata et al. 2011). The brain’s IGF-1 bioactivity and bioavailability are controlled by the IGF-binding protein (IGFBP) family. Furthermore, it has been suggested that brain IGFBPs participate not only in IGF-1 regulation but also in many brain processes, e.g., IGF-1-dependent neurogenesis, gliogenesis (Ajo et al. 2003), myelination, and synapse formation (Bunn et al. 2005). Data demonstrate that the concentrations of IGFBPs are area specific in the CNS (Ocrant 1991, 1993; Han et al. 1996). Among the six proteins, IGFBP-2 is believed to be the principal binding protein in the brain. Great importance is also ascribed to IGFBP-4, the only ‘classic’ binding protein, which does not exert any actions independently of IGF-1 (Mazerbourg et al. 2004; Ning et al. 2008). Some data have suggested that an appropriate balance between IGFBP-2 and IGFBP-4 is crucial for maintaining proper IGF-1 concentrations and biological activities.

IGF-1 expression in the brain is high during early organogenesis, and it diminishes after delivery (Ashpole et al. 2015). In adults, IGF-1 expression remains elevated only in areas with large projection neurons, such as the cerebellum, hippocampus, and cortex (Russo et al. 2005). Interestingly, the OBs are the regions where high levels of this protein and its receptor exist in both postnatal and adult brains. It has been postulated that high IGF-1 concentrations in this structure constitute a brain collateral “reservoir” for other brain areas, namely the hippocampus and frontal cortex (de Pablo and de la Rosa 1995; Freitas et al. 2013). Moreover, elevated IGF-1 concentrations are critical for embryonic and adult neurogenesis in the OB, especially in the processes of neuronal migration and positioning (Hurtado-Chong et al. 2009). In contrast, the experimental bulbectomy, thus lowering the IGF-1 level, has been established as a rodent model of depression. This procedure induces alterations in behavior, endocrine, immune, and neurotransmitter systems commonly observed in depressed patients (Kelly et al. 1997; Song and Leonard 2005). These data led to the suggestion that IGF-1 might act as an important factor in the onset of depression (Duman 2004). In fact, it has been demonstrated that, in rodents, IGF-1 can exert antidepressant-like effects after intracerebroventricular (ICV) or peripheral administration (Hoshaw et al. 2005; Duman et al. 2009; Basta-Kaim et al. 2014). Furthermore, IGF-1 could increase the basal level of serotonin in the ventral hippocampus 3 days after ICV administration, suggesting that IGF-1 could up-regulate serotonin levels in the brain (Hoshaw et al. 2008). On the other hand, chronic antidepressant treatment causes an increase in the hippocampal IGF-1 levels of rats and in the cerebrospinal fluid of humans (Khawaja et al. 2004). Based on the above-mentioned data, it may be suggested that there is an association of the IGF-1 system malfunction with the pathogenesis of depression.

To the best of our knowledge, there are no data concerning the regulation of the IGF-1 system in the OBs, the structures involved in the pathomechanism of depression. Therefore, the present study was designed to explore the mRNA expression and protein concentrations of IGF-1, as well as of IGF-1-binding protein levels (IGFBP-2, IGFBP-4, IGFBP-3, and IGFBP-6), in an animal model of depression. Moreover, we focused on IGF-1 receptor expression and the amounts of total and phosphorylated (active) intracellular IGF-1R subunit in OBs in adult rats that were prenatally stressed. In a subsequent set of experiments, the impact of the chronic administration of antidepressant drugs belonging to various chemical groups (imipramine, fluoxetine, and tianeptine) on the prenatal stress-evoked changes in the brain IGF-1 network was determined. In the present study, the widely accepted animal model of depression, based on the prenatal stress procedure, was chosen (Rao et al. 1999; Morley-Fletcher et al. 2003, 2004; Szymańska et al. 2009a; Budziszewska et al. 2010). This model differs from other stress-related models because the animals are exposed to stressful conditions in the prenatal phase. Therefore, the changes induced by stress are long lasting.

## Materials and Methods

### Animals

Sprague–Dawley rats (200–250 g upon arrival) obtained from Charles-Rivers (Sulzfeld, Germany) were kept under standard conditions (at room temperature of 23 °C, a 12/12 h light/dark cycle, and the lights on at 08:00 h), with food and water available ad libitum. Two weeks after arrival, vaginal smears were obtained daily from the female rats to determine the phase of the estrous cycle. On the proestrus day, the females were placed with males for 12 h and subsequently checked for the presence of sperm in vaginal smears. Pregnant females were randomly assigned to control and stress groups (*n* = 10 in each group). All of the experimental protocols were approved by the Committee for Laboratory Animal Welfare and Ethics of the Institute of Pharmacology, Polish Academy of Sciences, Cracow, and met the criteria of the International Council for Laboratory Animals and Guide for the Care and Use of Laboratory Animals. All efforts were made to minimize animal suffering and to reduce the number of animals used.

### Stress Procedure

The prenatal stress procedure was performed as previously described (Morley-Fletcher et al. 2003, 2004; Basta-Kaim et al. 2014; Szewczyk et al. 2014). Briefly, pregnant females were subjected to three stress sessions daily, beginning on the 14th day of pregnancy and continuing until delivery. At 09.00, 12.00 and 17.00 h, the rats were placed in plastic cylinders (7 × 12 cm) and illuminated with strong light (150 W) for 45 min. Control pregnant females were left undisturbed in their home cages. Only offspring from litters containing 10–12 pups with similar numbers of males and females were kept. Male offspring were selected from 21-day-old litters for the experiment. Twenty-four animals per group (control and prenatally stressed) were used for the experiments. They were housed in groups of four animals per cage (one or two animals from each litter) under standard conditions. At 3 months of age, the first behavioral verification was conducted.

### Forced Swim Test (FST)

As before for verification of the animal model of depression, the forced swim test (FST, Porsolt test) was conducted according to the method previously described (Detke et al. 1995; Basta-Kaim et al. 2014). Briefly, the animals were individually subjected to two trials, during which they were forced to swim in a cylinder (40 cm high and 18 cm in diameter) filled with water (25 °C) to a height of 30 cm. There was a 24-h interval between the first and second trials. The first trial lasted 15 min, and the second trial lasted 5 min. The total durations of immobility, mobility (swimming) and climbing were measured throughout the second trial (Porsolt et al. 1978; Detke et al. 1995).

### Antidepressant Drug Administration

**Fig. 1:**
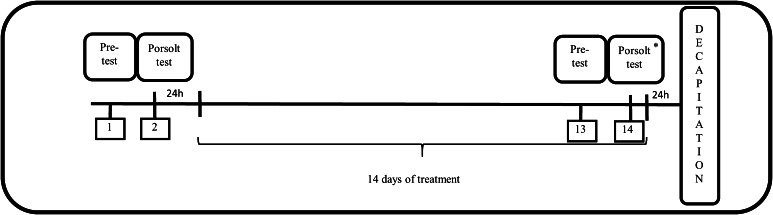
Schematic diagram representing the schedule of the experiment. *2 h after pretest (13th day) or Porsolt (14th day) tests animals were treated with the antidepressants

After the behavioral verification, the control and prenatally stressed offspring were randomly divided into 8 experimental groups (CONTROL + Veh, CONTROL + Imi, CONTROL + Flu, CONTROL + Tia, STRESS + Veh, STRESS + Imi, STRESS + Flu, and STRESS + Tia; 6 animals per group) and were treated for 14 days with antidepressant drugs. Imipramine (Imi; Sigma Aldrich, St. Louis, MO, USA), fluoxetine (Flu; Eli Lilly, Indianapolis, USA), or tianeptine (Tia; Tocris Bioscience, United Kingdom) were injected intraperitoneally, once daily between 09.00 and 10.00 h at a dose of 10 mg/kg in a volume of 2 ml/kg (diluted in 0.9 % saline). The CONTROL + VEH and STRESS + VEH groups received 0.9 % saline (Polpharma, Poland) in a volume of 2 ml/kg b.w. In the last 2 days of antidepressants treatment, the behavioral parameters in the forced swim test were measured (Fig. 1). The behavioral study was not blinded.

### Tissue Collection

Twenty-four hours after the last injection of antidepressant drugs, the animals were sacrificed under non-stress conditions by rapid decapitation. The OBs were dissected onto ice-cold glass plates, and tissues were frozen on dry ice and stored at −80 °C (for ELISA and western blot assays) or at −20 °C prior to total RNA extraction.

### Sample Preparation

The preparation of whole cell extracts was conducted according to the method described earlier (Budziszewska et al. 2010; Basta-Kaim et al. 2014). Briefly, the tissues were homogenized using an TissueLyzer II (Qiagen, Hilden Germany) in ice-cold radio-immunoprecipitation assay (RIPA) buffer, containing 100 μl of Phosphatase Inhibitor Cocktail 1 and 2, 100 μl of Protease Inhibitor Cocktail, 50 μl of phenylmethanesulfonyl fluoride (PMSF), and sodium orthovanadate (OVS) for a total volume of 500 µl (all reagents, Sigma Aldrich, St. Louis, MO, USA). The samples were shaken in an ice bath for 30 min and centrifuged at 14,000 rpm for 20 min at 4 °C. The supernatants were collected. The protein concentrations in the lysates were determined by the method of Lowry et al. (1951). The concentrations of cell extracts were standardized by dilution with lysis buffer to the lowest obtained protein concentration.

### Enzyme-Linked Immunosorbent Assay (ELISA)

The concentrations of IGF-1 and IGF-binding proteins (IGFBP-2; IGFBP-3; IGFBP-4; IGFBP-6) in the OB supernatants were determined by ELISA, using commercially available kits (IGF-1, IGFBP-2: all Mediagnost, Reutlingen, Germany; IGFBP-4, IGFBP-6: all USCNK Life Science Inc., People’s Republic of China; IGFBP-3: DEMEDITEC Diagnostics GmbH, Kiel-Wellsee, Germany), according to the manufacturers’ instructions. In case of IGF-1, total levels of IGF-1 (both free and bound) were determined.

Briefly, 50 μl of the standards or samples were dispensed into 96 wells coated with rat IGF-1, IGFBP-2, IGFBP-3, IGFBP-4, or IGFBP-6 antibody and were incubated at room temperature. After extensive washing, 100 μl of streptavidin-HRP was added, and the standards and samples were incubated for 30 min. 3,3′,5,5′-tetramethylbenzidine (TMB) (100 μl/well) was used as the chromogen for the colorimetric assay. The reaction was stopped after 10 min by adding 100 μl/well of stop solution, the absorbance was determined using the Infinite 200 PRO Detector (TECAN, Mannedorf, Switzerland) system set to 450 nm. The sensitivity of determination for each protein was as follows: IGF-1: 0.029 ng/ml; IGFBP-2: 0.01 ng/ml; IGFBP-3: 0.018 ng/ml; IGFBP-4: 0.148 ng/ml; and IGFBP-6: 0.064 ng/ml. For all of the study intra- and inter-assays, the coefficients of variation were always <7.5 and <10 %, respectively. Positive controls for each assay were provided by the manufacturers.

### Western Blot

The Western blot method was described by Basta-Kaim et al. (2011). Briefly, the cell lysates were mixed with a buffer (100 mM Tris–HCl, 4 % SDS, 20 % glycerol, 10 % 2-mercaptoethanol, 0.005 % bromophenol blue, pH 6.8) and boiled for 3 min before loading onto the gel. The proteins were separated by SDS-PAGE (4 % stacking gel and 10 % resolving gel) under constant voltage (90 V in stacking gel and 120 V in resolving gel) and were transferred electrophoretically onto the PVDF membrane (Roche Diagnostic, Germany) at 70 V for 1 h and 20 min. Membranes were washed twice with Tris-buffered saline (TBS), pH 7.5, blocked in 5 % nonfat milk for 1 h at room temperature, and incubated overnight at 4 °C with the appropriate primary antibody (anti-IGF-1 receptor beta (sc-713, Santa Cruz Biotechnology, Texas, USA) or anti-phospho-IGF-1 receptor antibody (ab85625, Abcam, USA) in 1 % nonfat milk solution. The blots were washed twice with TBS containing 0.1 % Tween-20 (TBST), twice with a 1 % blocking solution in TBS and then were incubated with a horseradish peroxidase-linked secondary antibody (sc-2030, Santa Cruz Biotechnology, Texas, USA) for 1 h at room temperature. Subsequently, the membranes were washed four times with large volumes of TBST, and the immunoblots were visualized with a chemiluminescence detection kit (Roche Diagnostic, Germany). β-actin (sc-47778, Santa Cruz Biotechnology, Texas, USA) levels were used for normalization. The semi-quantitative analysis of band intensity was performed using the LAS-4000 image analyzer and Multi Gauge software (FujiFilm, Japan).

### Real-Time Polymerase Chain Reaction (RT-PCR)

Freshly isolated OBs tissue samples were immediately placed in RNALater^®^ solution (Applied Biosystems, USA) and stored at −20 °C prior to total RNA extraction.

Total RNA was extracted using the RNeasy Mini Kit (Qiagen, Hilden, Germany) following the manufacturer’s instructions. RNA concentrations were determined using a NanoDrop spectrophotometer (ND/1000 UV/Vis; Thermo Fisher NanoDrop, USA). Identical amounts of RNA (1 µg) were reverse transcribed into cDNA using a commercial RT-PCR kit (Applied Biosystem, USA) according to the manufacturer’s instructions.

Real-time PCR was performed using TaqMan probes and primers for genes coding the following: IGF-1 (Rn00710306_m1), IGF-1R (Rn00583837_m1), and FastStart Universal Probe Master (Rox) mix (Roche, Germany). Amplification was performed in a total volume of 20 µl of mixture, containing 1× FastStart Universal Probe Master (Rox) mix (Roche, Germany), 50 ng of cDNA used as a PCR template, 900 nM TaqMan forward and reverse primers, and a 250 nM hydrolysis probe labeled with the fluorescent reporter dye FAM at the 5′-end, and quenching dye at the 3′-end. The thermal cycling conditions were as follows: 2 min at 50 °C and 10 min at 95 °C followed by 40 cycles at 95 °C for 15 s and 1 min at 60 °C. Samples were run in duplicate. The threshold value (*C*_t_) for each sample was set in the exponential phase of PCR, and the ΔΔ*C*_t_ method was used for data analysis (Applied BioSystems, United Kingdom). Beta-2-microglobulin (B2 M, Rn00560865_m1) was used as the reference gene.

### Statistical Analysis

The outcomes of the behavioral studies are presented as the means ± SEMs. The data obtained in the ELISA study are presented as weight units (ng) per milligram of protein ± SEMs; those for RT-PCR as the average fold ± SEM, and for Western blotting, the results are presented as percentage of the control ± SEM. The normality of variable distribution and homogeneity of variances were checked by the Shapiro–Wilk test and Levene’s test, respectively. The significance of the differences between the means was evaluated by one- or two-way analysis of variance (ANOVA), with Duncan’s post hoc test if appropriate. A value of *p* < 0.05 was considered statistically significant. All of the statistical analyses were performed using Statistica software, version 10.0 (Statsoft, Tulsa, USA).

## Results

### The Impact of Prenatal Stress on the Immobility, Swimming, and Climbing Time on the Forced Swim Test

To evaluate depression-like behavior in rats, we performed the forced swim test (Porsolt test). We confirmed that prenatal stress caused significantly prolonged immobility time (in seconds) (control—213.52 ± 8.92, stress—263.45 ± 7.33; *F*_1,46_ = 288.92; *p* < 0.05) and shortened swimming time (in seconds) (control—86.47 ± 8.92, stress—41.45 ± 7.33; *F*_1,46_ = 398.73; *p* < 0.05), as well as climbing time (in seconds) (control—71.79 ± 9.80 stress—39.14 ± 6.51; *F*_1,46_ = 224.37; *p* < 0.05).

### The Impact of Antidepressant Drug Treatment on the Immobility, Swimming, and Climbing Time in the Forced Swim Test

**Fig. 2:**
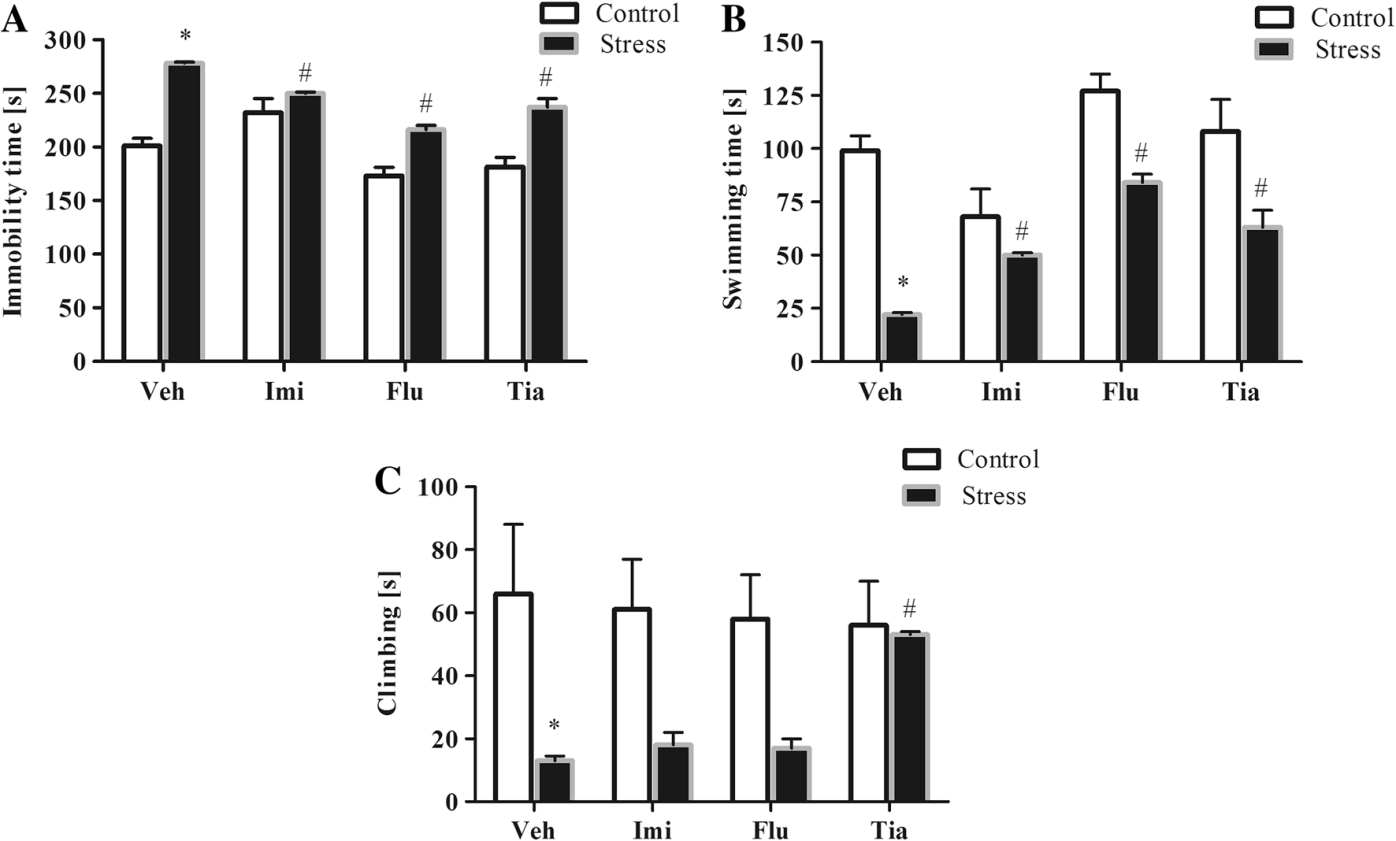
The effects of prenatal stress and chronic antidepressant drug administration on the immobility (**a**), mobility (**b**), and climbing time (**c**) (in seconds) in the forced swim test. The data are presented as the means ± SEMs, with *n* = 5–6 for each group. *p ≤ 0.05 versus control Veh group; ^#^ *p* ≤ 0.05 versus prenatally stressed Veh group. ANOVA (two-way), followed by Duncan’s test. *Veh* vehicle, *Imi* imipramine, *Flu* fluoxetine, *Tia* tianeptine

Next, to determine whether chronic imipramine, fluoxetine, or tianeptine administration affected the behavioral changes evoked by the prenatal stress, we assessed the Porsolt test again. As previously, we observed in prenatally stressed rats increase in the immobility (*F*_1,39_ = 90.40; *p* < 0.05) and decrease in swimming (*F*_1,39_ = 57.34; *p* < 0.05) and climbing (*F*_1,39_ = 17.12; *p* < 0.05) times, which confirmed that the changes induced by stress were long lasting (Fig. 2a*–*c). Furthermore, we revealed a significant effect of drugs (*F*_3,39_ = 20.32; *p* < 0.05; Fig. 2a) and the Stress × Drug Interaction (*F*_3,39_ = 5.48; *p* < 0.05; Fig. 2a) on the immobility time. Post hoc comparisons revealed that imipramine (*p* < 0.05), fluoxetine (*p* < 0.05) and tianeptine (*p* < 0.05) shortened immobility time in prenatally stressed offspring. Also for swimming time, we observed a significant effect of drugs (*F*_3,39_ = 13.49; *p* < 0.05; Fig. 2b) and the Stress × Drug Interaction (*F*_3,39_ = 3.76; *p* < 0.05; Fig. 2b). Moreover, post hoc comparisons revealed that imipramine (*p* < 0.05), fluoxetine (*p* < 0.05), and tianeptine (*p* < 0.05) extended the swimming time in stressed offspring. In the case of the climbing time (Fig. 2c), we observed that only tianeptine (*p* < 0.05) prolonged the climbing time in prenatally stressed rats.

### The Impact of Prenatal Stress and Chronic Antidepressant Drug Administration on the mRNA Expression and Protein Levels of IGF-1 in the OBs

**Fig. 3:**
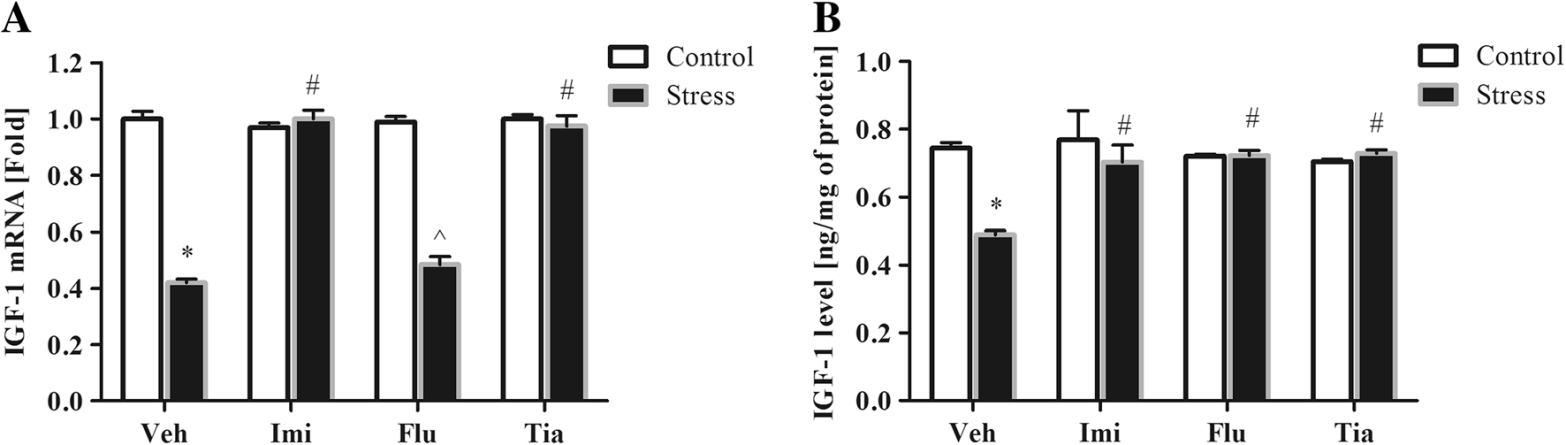
The effects of prenatal stress and imipramine, fluoxetine, or tianeptine administration on IGF-1 mRNA expression (**a**) and IGF-1 levels (**b**) in the olfactory bulbs. mRNA expression is presented as the average fold ± SEM and the protein level as ng/mg of protein (mean ± SEM). *n* = 5–6 for each group, *p ≤ 0.05 versus control Veh group; ^*p* ≤ 0.05 versus control Flu group; ^#^ *p* ≤ 0.05 versus prenatally stressed Veh group. ANOVA (two-way), followed by Duncan’s test. *Veh* vehicle, *Imi* imipramine, *Flu* fluoxetine, *Tia* tianeptine

Examination of the samples derived from the OBs revealed a significant down-regulation in the IGF-1 mRNA expression (*F*_1,34_ = 172.53; *p* < 0.05) in prenatally stressed rats compared with the controls (unstressed animals) (Fig. 3a). Moreover, ANOVA showed a significant decrease in the IGF-1 protein concentrations in prenatally stressed offspring (*F*_1,32_ = 8.09; *p* < 0.05) (Fig. 3b).

We also examined the effects of chronic antidepressant treatment on the mRNA expression and protein concentrations of IGF-1 in the OBs. As illustrated in Fig. 3, ANOVA showed a significant effect of drugs (*F*_3,34_ = 58.65; *p* < 0.05) and the Stress × Drug Interaction on IGF-1 mRNA expression (*F*_3,34_ = 64.27; *p* < 0.05). We also noted a significant effect of drugs (*F*_3,32_ = 4.20; *p* < 0.05) and the Stress × Drug interaction on IGF-1 protein levels (*F*_3,32_ = 5.76; *p* < 0.05) (Fig. 3b). Post hoc comparisons revealed that chronic treatment with imipramine (*p* < 0.05), and tianeptine (*p* < 0.05) increased both the mRNA expression and protein concentrations of IGF-1, but fluoxetine (*p* < 0.05) only IGF-1 level in the OBs of prenatally stressed offspring.

### The Impact of Prenatal Stress and Chronic Antidepressant Drug Administration on the IGF-1 Binding Proteins Levels in OBs

To determine whether prenatal stress affected other proteins in the IGF-1 family, we examined the levels of IGF-1-binding proteins, i.e., IGFBP-2, -4, -3, and -6.

**Fig. 4:**
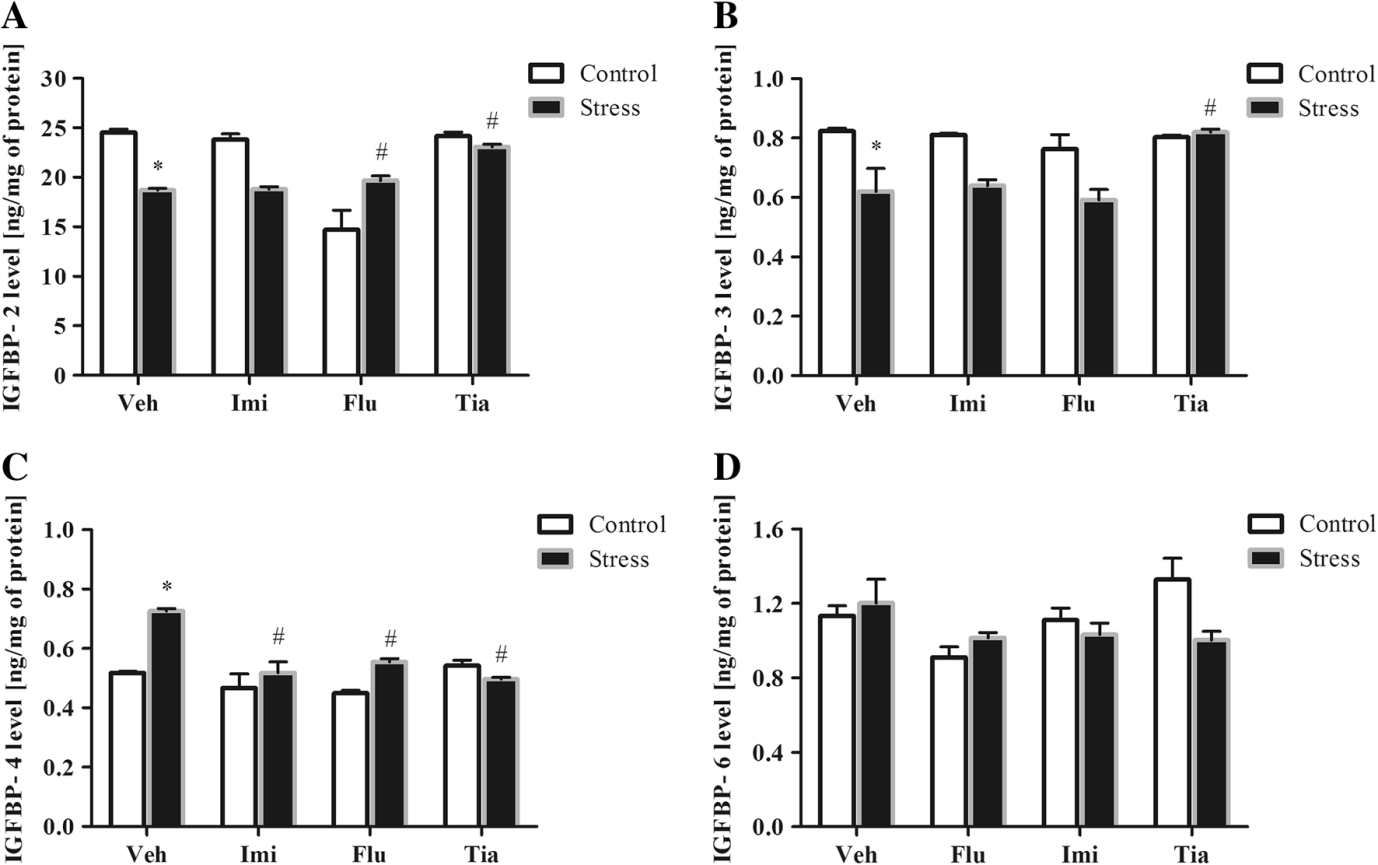
The effects of prenatal stress and imipramine, fluoxetine, or tianeptine administration on the levels of IGFBP-2 (**a**), IGFBP-3 (**b**), IGFBP-4 (**c**), and IGFBP-6 (**d**) in the olfactory bulbs. The results are presented as ng per mg of protein (mean ± SEM). *n* = 5–6 for each group, *p ≤ 0.05 versus control Veh group; ^#^ *p* ≤ 0.05 versus prenatally stressed Veh group. ANOVA (two-way) followed by Duncan’s test. *Veh* vehicle, *Imi* imipramine, *Flu* fluoxetine, *Tia* tianeptine

The results of ANOVA showed a significant decrease in IGFBP-2 (main binding protein expressed in the brain) level of prenatally stressed offspring (*F*_1,32_ = 58.04; *p* < 0.05). We also noted a significant effect of drugs (*F*_3,32_ = 141.74; *p* < 0.05) and a significant Stress × Drug interaction (*F*_3,32_ = 118.86; *p* < 0.05). For the first time, we demonstrated that chronic administration of fluoxetine (*p* < 0.05) and tianeptine (*p* < 0.05) statistically significantly increased IGFBP-2 levels in the OBs of prenatally stressed animals (Fig. 4a).

In the next set of experiments, we found that the level of IGFBP-4, which operates in the brain mainly as an inhibitor of IGF-1 signaling, was significantly elevated in the OBs of prenatally stressed rats (*F*_1,32_ = 31.00; *p* < 0.05). Moreover, ANOVA showed a significant effect of drugs (*F*_3,32_ = 21.42; *p* < 0.005) and a Stress × Drug interaction (*F*_3,32_ = 18.95; *p* < 0.05). Post hoc comparisons revealed that all of the antidepressants—fluoxetine (*p* < 0.05), tianeptine (*p* < 0.05), and imipramine (*p* < 0.05)—decreased the IGFBP-4 level in prenatally stressed offspring (Fig. 4c).

Furthermore, our study demonstrated that the prenatal stress procedure reduced the IGFBP-3 concentration (*F*_1,32_ = 72.14; *p* < 0.05). Among the antidepressants tested, post hoc comparison showed that only tianeptine (*p* < 0.05) increased IGFBP-3 levels in the OBs of prenatally stressed offspring (Fig. 4b). In contrast, no changes in IGFBP-6 levels were observed after the prenatal stress procedure (*F*_1,32_ = 1.12; ns) and antidepressant drug administration (*F*_3,32_ = 3.24; ns) (Fig. 4d).

### The Impact of Prenatal Stress and Chronic Antidepressant Drug Administration on the mRNA Expression and Phosphorylation Level of IGF-1 Receptor in OBs

**Fig. 5:**
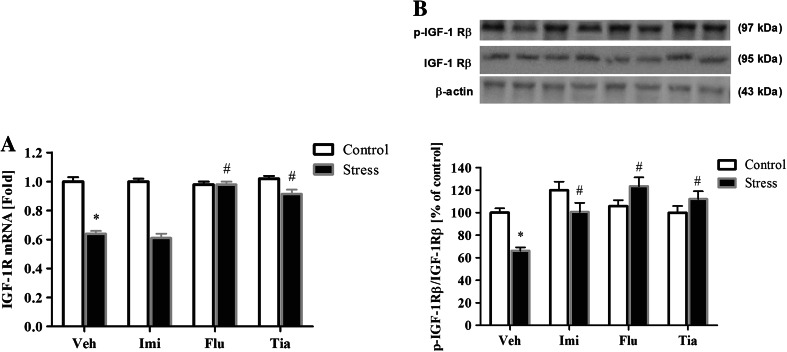
The effects of prenatal stress and imipramine, fluoxetine, or tianeptine administration on IGF-1R mRNA expression (**a**) and on the ratio of the phosphorylated (active) form of the β subunit to the total β subunit (**b**) in the olfactory bulbs. mRNA expression is presented as the average fold ± SEM; the protein levels are normalized to β-actin and presented as the % of control ± SEM. *n* = 5–6 for each group, *p ≤ 0.05 versus control Veh group; ^#^ *p* ≤ 0.05 versus prenatally stressed Veh group. ANOVA (two-way), followed by Duncan’s test. *Veh* vehicle, *Imi* imipramine, *Flu* fluoxetine, *Tia* tianeptine

The results of RT-PCR analysis revealed that IGF-1R gene expression was statistically significantly decreased (*F*_1,34_ = 81.93; *p* < 0.05) in animals subjected to prenatal stress (Fig. 5a). Next, ANOVA showed a significant Drug × Stress interaction regarding IGF-1R mRNA expression (*F*_3,34_ = 13.67; *p* < 0.05). Post hoc comparisons revealed that chronic administration of fluoxetine (*p* < 0.05) and tianeptine (*p* < 0.05) increased the IGF-1R mRNA expression in prenatally stressed offspring. On the other hand, imipramine had no effect on the IGF-1R mRNA expression (Fig. 5a).

Moreover, prenatal stress significantly decreased the ratio of the phosphorylated form of the β subunit to the total IGF-1 R β subunit (*F*_1,34_ = 4.41; *p* < 0.05). We also noted significant effects of drug (*F*_3,34_ = 18.89; *p* < 0.05) and the Stress × Drug Interaction (*F*_3,34_ = 15.17; *p* < 0.05) (Fig. 5b). Post hoc comparisons revealed that fluoxetine (*p* < 0.05), tianeptine (*p* < 0.05), and imipramine (*p* < 0.05) normalized the IGF-1 receptor phosphorylation in stressed animals (Fig. 5b).

## Discussion

Present studies confirmed our observations indicating behavioral disturbances in the offspring of rat dams that were stressed during pregnancy (Morley-Fletcher et al. 2003, 2004; Szymanska et al. 2009a, b; Budziszewska et al. 2010; Basta-Kaim et al. 2014). Prenatally stressed rats exhibit not only an increase in immobility time but also decreases in swimming and climbing behaviors, as shown in the modified Porsolt test. Moreover, the current study showed that imipramine, fluoxetine, and tianeptine eliminated stress-evoked changes in immobility and swimming behavior. Additionally, chronic tianeptine administration prolonged the climbing time in the modified Porsolt test in adult offspring. The effect of tianeptine may be due to its complex action including the unique properties on the serotoninergic system, by increasing reuptake and accelerate of serotonin turnover in the synapse (Uzbekov 2009). Moreover, tianeptine may modulate the glutamatergic and GABAergic systems (McEwen et al. 2010), which as we previously demonstrated are impaired in prenatally stressed animals (Sowa et al. 2015). On the other hand, the sensitivity of detailed assessment of animal’s behavior in response to tianeptine administration in the Porsolt’s test should be also considered.

Besides the behavioral alterations, this report for the first time characterized the impact of chronic antidepressant treatment on the IGF-1 system responsible for the biological function of IGF-1 in the OBs of an animal model of depression. Our study showed that, evoked by prenatal stress procedures, disturbances in IGF-1 and the IGFBPs and the phosphorylation of IGF-1 receptor were normalized by chronic antidepressant treatment.

Considering that IGF-1 is a particular factor regulating cell growth, differentiation, exhibiting neurotrophic, neurogenic and neuroprotective actions (Russo et al. 2005), it has been postulated that IGF-1 network disturbances might be crucial to the pathomechanism of affective disorders.

To the best of our knowledge, the impact of prenatal stress on the expression of proteins belonging to the IGF-1 family in the OBs has not been evaluated. Therefore, as observed in our study, the decreases in IGF-1 mRNA expression and protein concentrations in OBs may be discussed only in comparison with animal studies that defined IGF-1 expression in other brain areas important to the pathogenesis of depression, especially the frontal cortex and hippocampus. In fact, our previous results demonstrated that the prenatal stress procedure led to a decrease in the IGF-1 concentration in the frontal cortex and hippocampus of adult offspring (Basta-Kaim et al. 2014), which might suggest that the IGF-1 changes in the current model of depression occurred independent of the examined brain area. It is worth emphasizing that we also demonstrated that behavioral disturbances in prenatally stressed animals reversed the intracerebroventricular administration of IGF-1 (Basta-Kaim et al. 2014). Based on these observations, it could be suggested that, as observed in the present study, also diminished IGF-1 levels in OBs might be at least partially responsible for depressive-like behavior in stressed rats.

The mechanism responsible for brain IGF-1 regulation is fulfilled by IGF-binding proteins belonging to the IGF family. They act as carrier proteins for IGFs, regulate IGF-1 transport, control its diffusion and efflux from the vascular space, increase its half-life, and modulate the interaction of IGF-1 with its receptors (Russo et al. 1997; Firth and Baxter 2002).

Particularly noteworthy is that we demonstrated changes in IGFBP levels in the OBs, such as decreases in IGFBP-2 and IGFBP-3, as well as an increase in IGFBP-4 concentrations. The OBs under normal conditions are rich in locally expressed components of the IGF system, including IGFBP-2, which is secreted by brain-resident neural and glial cells and regulates the transport and target of IGFs to the brain areas and prolongs the half-life of IGF-1 (Bezchlibnyk et al. 2006; Chesik et al. 2007). Data showed that IGF-I complexes with IGFBP-2 might promote neurogenesis in adult animals and that this process was inhibited by the IGFBP-2 antibody (Brooker et al. 2000). Moreover, IGFBP-2 can bind to components of the extracellular matrix and concentrate IGF-1 near its receptor, thus enhancing IGF-1 activity (Bourner et al. 1992; Bezchlibnyk et al. 2006). Therefore, it might be postulated that the dampened IGFBP-2 levels observed in the OBs of adult male rats after the prenatal stress procedure might be responsible not only for the reduction in IGF-1 levels but also the interaction of IGF-1 with IGF-1 receptor.

Our previous data obtained from the hippocampus and frontal cortex showed decreases in the concentrations of IGFBP-2, as well as enhanced levels of IGFBP-4 in prenatally stressed animals (Basta-Kaim et al. 2014). In the present study in the OBs, we also demonstrated up-regulation of IGFBP-4, a protein involved in cell growth, development and common neuromodulation in the frontal cortex, hippocampus and OBs (Werther et al. 1998; Russo et al. 2005). Because most of the previous studies indicated an inhibitory role of IGFBP-4 on the IGF-1 system both in vivo and in vitro (Mazerbourg et al. 2004; Praveen Kumar et al. 2014), it might be suggested that IGFBP-4 up-regulation in prenatally stressed rats might be responsible for diminished IGF-1 levels and the suppression of IGF-1 signaling.

The second main finding of our study was the observation that chronic imipramine, fluoxetine, and tianeptine administration up-regulated IGF-1 expression in the OBs of prenatally stressed rats. Moreover, antidepressants affected the malfunction of the IGFBP network; imipramine, fluoxetine, and tianeptine attenuated IGFBP-4 levels, while the IGFBP-2 concentration was increased by fluoxetine and tianeptine. The outcomes of the present work showed that chronic antidepressant treatment restored the correct balance between IGFBP-2 and IGFBP-4, the levels of which determine proper biological action of IGF-1. Furthermore tianeptine treatment enhanced IGFBP-3 concentrations diminished by prenatal stress, which are important in the control of neurogenesis, gliogenesis, myelination, and synaptic formation (Ajo et al. 2003; Bunn et al. 2005; Kalluri and Dempsey 2011). In contrast, none of the antidepressant drugs affected the IGFBP-6 level.

It should be emphasized that there are no data concerning the impact of chronic antidepressant administration on the IGF-1 network in the OB. However, in some brain areas, antidepressants, especially fluoxetine, potentiate IGF-I expression (Khawaja et al. 2004) and increase its concentrations in the cerebrospinal fluid (Schilling et al. 2011). Although there is little information about the involvement of antidepressant drugs on IGF-1 activity, it has been found that fluoxetine administration affects the IGF-1 system in the cortex and hippocampus differently. After chronic fluoxetine treatment, the hippocampus displayed IGF-1 down-regulation, while the frontal cortex IGF-1 level tended to be up-regulated (Grunbaum-Novak et al. 2008). Moreover, a specific antibody against the IGF-I receptor or an antiserum raised against IGF-I reversed this antidepressant-evoked potentiation (Paslakis et al. 2012). Thus, the studies conducted so far have indicated that the effect of antidepressant drugs on IGF-1 depends not only on the treatment duration and brain structure but also; they may also differently affect IGF-1 mRNA expression and concentration, like in our studies fluoxetine did. Unequivocal determination of mechanisms of those discrepancies requires further studies.

Our present results indicated that chronic fluoxetine treatment not only up-regulated IGF-1 levels but also enhanced the IGF-1 beta-subunit receptor phosphorylation, which was diminished in the OBs of prenatally stressed offspring. The same effect after chronic tianeptine and imipramine treatment has been observed.

The IGF-1 receptor is expressed in both the OB and olfactory epithelium; therefore, some data have suggested an important role in the bulbo-epithelial interactions of the IGF-1 family in the olfactory system (Matsui et al. 2014). Because antidepressant treatment promotes neuronal plasticity, including neurogenesis and neuronal maturation, an increase in IGF-1 receptor phosphorylation in the OB, particularly induced by fluoxetine and tianeptine, might be relevant to the antidepressant action. But it is also possible that chronic treatment with antidepressants by normalization of both the immune system and HPA axis activity (Pariante and Miller 2001; Pariante et al. 2004) increases IGF-1 expression and IGF-1 receptor phosphorylation (Castren 2004).

Because our study demonstrated IGF-1 system malfunction in prenatally stressed rats, it could be postulated that biological IGF-1 properties in the OB, such as myelination of neuronal axons, formation of synaptic networks, synaptic plasticity, survival, longevity of cells in developing and adult neurons, and cognitive functions, are disturbed. Moreover, because the data demonstrated that IGF-1 plays an important role during neurogenesis, including its involvement in regulating radial neuronal migration and positioning within the OB, as well as in the incorporation of newly formed neurons from the subventricular zone (SVZ) into the OB, the prenatal stress procedure might disrupt their course (Hurtado-Chong et al. 2009; Whitman and Greer 2009). Finally, because the OBs have connections with other brain regions, mainly the olfactory-limbic circuit, disturbances in the olfactory IGF-1 system of adult rats might affect reorganization processes in limbic and cortical areas, and these disturbances appear to be responsible for behavioral abnormalities (Zueger et al. 2005; Machado et al. 2012). Thus, the beneficial effect of antidepressant drugs on behavioral disturbances might result from their impact on the IGF-1 network, not only in the OBs but also in the limbic system remaining dependent of them.

## Conclusions

In conclusion, our study provided the first evidence that stress during pregnancy led not only to behavioral disturbances but also to malfunction of the IGF-1 network in OBs. Furthermore, it was found that the beneficial effects of chronic antidepressant drug treatment resulted not only from the potentiation of IGF-1 receptor expression and phosphorylation but also from the normalization of the IGF-1 level, probably via its impact on the IGF-1 binding protein network in adult male rats. It can be suggested that the IGFBP system might be a potential target for antidepressant drug treatment, but these hypotheses require further investigations.

## Abbreviations

ANOVA: Analysis of variance
BDNF: Brain-derived neurotrophic factor
CNS: Central nervous system
ELISA: Enzyme-linked immunosorbent assay
Flu: Fluoxetine
IGF-1: Insulin-like growth factor-1
IGF-1R: Insulin-like growth factor-1 receptor
IGFBP: Insulin-like growth factor binding protein
Imi: Imipramine
OBs: Olfactory bulbs
OVS: Sodium orthovanadate
PMSF: Phenylmethanesulfonyl fluoride
PVDF: Polyvinylidene fluoride
SEM: Standard error of the mean
SVZ: Subventricular zone
TBS: Tris-buffered saline
Tia: Tianeptine
TMB: 3,3′,5,5′-Tetramethylbenzidine
Veh: Vehicle
